# The histopathological spectrum of malignant hyperthermia and rhabdomyolysis due to *RYR1* mutations

**DOI:** 10.1007/s00415-019-09209-z

**Published:** 2019-02-20

**Authors:** G. J. Knuiman, B. Küsters, L. Eshuis, M. Snoeck, M. Lammens, L. Heytens, W. De Ridder, J. Baets, R. S. Scalco, R. Quinlivan, J. Holton, I. Bodi, E. Wraige, A. Radunovic, C. von Landenberg, J. Reimann, E.-J. Kamsteeg, C. Sewry, H. Jungbluth, N. C. Voermans

**Affiliations:** 1 Department of Pathology, Radboud University Medical Centre, Nijmegen, The Netherlands; 2 National MH Investigation Unit, Department of Anaesthesiology, Canisius Wilhelmina Hospital, Nijmegen, The Netherlands; 3 Department of Pathology, Antwerp University Hospital, University of Antwerp, Edegem, Belgium; 4 Malignant Hyperthermia Research Unit, University of Antwerp, Antwerp, Belgium; 5 Neurogenetics Group, Center for Molecular Neurology, VIB, Antwerp, Belgium; 6 Laboratory of Neuromuscular Pathology, Institute Born-Bunge, University of Antwerp, Antwerp, Belgium; 7 Department of Neurology, Neuromuscular Reference Centre, Antwerp University Hospital, Antwerp, Belgium; 8 MRC Centre for Neuromuscular Diseases, Institute of Neurology, University College London, London, UK; 9 Department of Paediatric Neurology, Neuromuscular Service, Evelina Children’s Hospital, Guy’s and St Thomas’ Hospital NHS Foundation Trust, London, UK; 10 Barts Neuromuscular Diseases Centre, Royal London Hospital, London, UK; 11 Muscle Lab, Department of Neurology, University of Bonn Medical Centre, Bonn, Germany; 12 Department of Human Genetics, Radboud University Medical Centre, Nijmegen, The Netherlands; 13 Dubowitz Neuromuscular Centre, UCL Institute of Child Health and Great Ormond Street Hospital for Children, London, UK; 14 Muscle Signalling Section, Randall Division for Cell and Molecular Biophysics, King’s College, London, UK; 15 Department of Basic and Clinical Neuroscience, King’s College, IoPPN, London, UK; 16 Department of Neurology, Radboud University Medical Centre, Nijmegen, The Netherlands

**Keywords:** RYR1, RyR1, Rhabdomyolysis, Malignant hyperthermia (MH), Muscle biopsy, Histology.

## Abstract

**Objective:**

The histopathological features of malignant hyperthermia (MH) and non-anaesthetic (mostly exertional) rhabdomyolysis (RM) due to *RYR1* mutations have only been reported in a few cases.

**Methods:**

We performed a retrospective multi-centre cohort study focussing on the histopathological features of patients with MH or RM due to *RYR1* mutations (1987–2017). All muscle biopsies were reviewed by a neuromuscular pathologist. Additional morphometric and electron microscopic analysis were performed where possible.

**Results:**

Through the six participating centres we identified 50 patients from 46 families, including patients with MH (*n* = 31) and RM (*n* = 19). Overall, the biopsy of 90% of patients showed one or more myopathic features including: increased fibre size variability (*n* = 44), increase in the number of fibres with internal nuclei (*n* = 30), and type I fibre predominance (*n* = 13). Abnormalities on oxidative staining, generally considered to be more specifically associated with *RYR1*-related congenital myopathies, were observed in 52%, and included unevenness (*n* = 24), central cores (*n* = 7) and multi-minicores (*n* = 3). Apart from oxidative staining abnormalities more frequently observed in MH patients, the histopathological spectrum was similar between the two groups. There was no correlation between the presence of cores and the occurrence of clinically detectable weakness or presence of (likely) pathogenic variants.

**Conclusions:**

Patients with *RYR1*-related MH and RM exhibit a similar histopathological spectrum, ranging from mild myopathic changes to cores and other features typical of *RYR1*-related congenital myopathies. Suggestive histopathological features may support *RYR1* involvement, also in cases where the in vitro contracture test is not informative.

**Electronic supplementary material:**

The online version of this article (10.1007/s00415-019-09209-z) contains supplementary material, which is available to authorized users.

## Introduction

Mutations in the skeletal muscle ryanodine receptor (*RYR1*) gene are amongst the most common causes of non-dystrophic neuromuscular disorders [1], and associated with a wide clinico-pathological spectrum. *RYR1* encodes RyR1, the principal sarcoplasmic reticulum calcium release channel with a crucial role in excitation–contraction coupling. Dominant *RYR1* mutations were originally implicated in two distinct but occasionally overlapping human disorders, Central Core Disease (CCD), a congenital myopathy named after the typical core structures with reduced oxidative stains on muscle biopsy, and the Malignant Hyperthermia Susceptibility (MHS) trait, a pharmacogenetic predisposition to potentially life-threatening episodes of muscle breakdown and metabolic decompensation in response to volatile anaesthetics and/or the neuromuscular blocking agent succinylcholine.

The last two decades have seen an ever-increasing expansion of the *RYR1*-associated phenotypic spectrum: in addition to dominantly inherited CCD, other (mainly recessively inherited) congenital myopathies named after the predominant finding on muscle biopsy—Multi-minicore Disease (MmD) [2], Centronuclear Myopathy (CNM) [3], and Congenital Fibre Type Disproportion (CFTD)—have been described [4]. More recently, the clinical spectrum of predominantly MHS-associated *RYR1* mutations has also further expanded, and was found to include other episodic phenotypes such as exertional myalgia associated with rhabdomyolysis (RM) [5] and periodic paralysis [6, 7], but also a distinct late-onset axial myopathy [8, 9] in previously healthy individuals.

Whilst the histopathological spectrum of *RYR1*-associated congenital myopathies has been characterized extensively (for review: Jungbluth et al. [1]) [3, 4, 10–12], there is little information concerning the histopathological spectrum of MH- and RM-associated *RYR1* mutations. Moreover, many of the reports concerning histopathological features in MH and RM predate their genetic resolution [13–18]. The relative scarcity of MH-related histopathological data in particular may reflect that MH is essentially a clinical diagnosis, and that muscle biopsies in these patients are mainly performed for the purpose of a diagnostic halothane-caffeine in vitro contracture test (IVCT) rather than for primary histopathological evaluation. The few available reports suggest some overlap with the milder end of the *RYR1*-associated congenital myopathy spectrum, or even a normal muscle biopsy appearance [5, 19–21]. The only large retrospective study on histopathological changes in MH included 399 MHS patients, of whom 86 (22%) patients had histological abnormalities. However, only 226 (57%) patients were genetically tested, and of them, only 86 (38%) had a confirmed *RYR1* mutation [22].

In the present study, we, therefore, focused on genetically confirmed cases. We reviewed the muscle biopsy findings from 50 paediatric and adult patients presenting with *RYR1*-associated MH or RM. We have delineated the key histopathological features, also in comparison to the already recognized *RYR1*-associated congenital myopathy spectrum, and have tried to establish tentative genotype–phenotype correlations on the histopathological level. We have emphasized histopathological features that should raise the possibility of *RYR1* involvement, in particular in episodic phenotypes such as RM, a very common presentation with many potential genetic causes but often no specific clinical signs [23].

## Patients and methods

### Methods

We performed a retrospective multi-centre cohort study focussing on the histopathological features of children and adults with MH or RM in whom an *RYR1* mutation was detected and a diagnostic muscle biopsy had been performed (1983–2017; *RYR1* sequencing since 2006). RM was defined as an acute muscle breakdown in response to an external trigger [such as exercise, (recreational) drugs, medication, viral illness] associated with symptoms of myalgia, swelling, muscle weakness and/or myoglobinuria, reflected by a sudden rise (at least 10 times the upper normal limit for sex and ethnicity) and subsequent normalisation of CK levels. Biopsies had been taken at least 2 months after the MH or RM episode, except in patient 34 and 46, in whom the biopsy had been taken in the acute RM phase.

The study protocol was approved by the Radboud UMC Ethics Committee (2017-3903).

### Patients

Key clinical (but not histopathological) features from 34 patients included in this study have already been reported by Snoeck et al. and Dlamini at al. (Supplementary File 1) [5, 21]. In the current study, muscle biopsies were included for review from additional patients seen at tertiary MH and neuromuscular referral centres: Antwerp University Hospital (Belgium), University of Bonn Medical Centre (Germany), Radboud University Medical Centre (The Netherlands), Evelina Children’s Hospital, London, The National Hospital for Neurology and Neurosurgery (NHNN), and the Royal London Hospital, London (United Kingdom). Relatives carrying the familial *RYR1* mutation (with a positive IVCT) were also included. Patients were only included if the original muscle biopsy slides were available for (re)evaluation. The following data were collected: clinical diagnosis, presumed mode of inheritance, presence of muscle weakness, and result of IVCT. The IVCT had been performed (partly in the pre-genetic era) in patients who later proved to have a diagnostic MH mutation (see below), according to the European Malignant Hyperthermia Group (EMHG) protocol (https://www.emhg.org/testing-for-mh/2017/12/28/in-vitro-contracture-testing-ivct).

### Molecular genetic studies

The coding regions (exons 1–106) of the *RYR1* gene, including splice sites, were screened at the genomic level by standard Sanger sequencing or panel sequencing (RM panel since 2014 [23]). Haplotyping of unrelated patients carrying the recurrent *RYR1* mutation c.12861_12869dup (p.Thr4288_Ala4290dup) was carried out using a panel of highly polymorphic microsatellite repeat markers located in and around the *RYR1* locus [3, 5]. Until 2008, MH patients had first been tested by multiplex ligation-dependent probe amplification (MLPA) using two kits containing the first 27 functionally characterized diagnostic MH mutations (i.e., functionally fully characterized *RYR1* mutation recognized as MH causative; http://www.emhg.org/diagnostic-mutations) supplemented with six recurrent non-functionally characterized mutations [21]. Relatives were investigated for the presence of the familial mutations only. For all *RYR1* mutations it was checked whether they were diagnostic MH mutations according to the EMGH; if not, variants were classified according to current American College of Medical Genetics and Genomics (ACMG) guidelines as pathogenic, likely pathogenic, variant of undetermined significance (VUS) or likely benign [24, 25]. Results of other genetic tests in patients with RM were also collected.

### Histopathological studies

The neuromuscular pathologists (JK and BK at Radboudumc and JH at NHNN) reviewed the standard histological [hematoxylin and eosin, H&E, Gomori trichrome, periodic acid-Schiff (PAS), sudan black or oil red O] and histochemical [nicotinamide adenosine dinucleotide-tetrazolium reductase (NADH-TR); succinate dehydrogenase (SDH), ATPase, pre-incubated at pH 10.3, 4.6 and 4.3; cytochrome c oxidase, (COX) stains as available]. (In all cases at least a hematoxylin and eosin staining and an oxidative stain were available for review.) Cores and minicores were defined as previously reported [10]. Type 1 predominance was defined as ≥ 55% type 1 fibres [26]. Fibre type disproportion was qualitatively evaluated on the basis of the ATPase pre-incubated at a pH of 4.2-staining. Increased fibre size variation was defined as an increased ratio (> 2) between largest and smallest fiber size [26]. After individual evaluation of each biopsy, consensus was reached by open discussion. Electron microscopy reports were reviewed where available (*n* = 27; 54% of patients).

### Statistics

The difference in occurrence of specific histopathological features in patients with MH and RM were compared by a Chi-square test. The age difference between groups with and without cores was calculated by an independent *T* test.

## Results

### Patients and biopsies

We identified 50 patients from 46 families, including 31 MH cases (16 index patients and 15 first-degree adult relatives carrying the familial *RYR1* variant with a postivie IVCT who had volunteered to undergo the IVCT instead of the mostly paediatric index patient) and 19 individuals who had suffered from RM. One patient (patient 38) within the RM group had an initial episode of exertional RM followed by an MH event. Four pairs of related patients were included (patients 6 and 7, 15 and 16, 23 and 24 in the MH group, and 32 and 33 in the RM group). All RM patients had one or more features suggesting an underlying genetic cause [recurrent episodes of exertional rhabdomyolysis; hyperCKaemia persisting 8 weeks after the event; RM occurring after accustomed physical exercise; blood CK > 50 × upper limit of normal (> 10,000 ULN in female Caucasian patients); absence of drugs/medication/supplements that could explain the rhabdomyolysis; and/or other family members affected, or other exertional symptoms (cramps, myalgia)] [27] Recurrent episodes were reported in 9 of 13 patients. Needle or open biopsies were taken from the quadriceps muscle (mostly vastus lateralis) unless specified otherwise (Supplementary File 2).

Clinical findings are summarized in Table 1. There was predominance of male patients (74%; 20/31 MH and 17/19 RM). Likely triggers for RM were: heat (*n* = 2), infection (*n* = 2) and exertion (*n* = 17); in addition, one RM patient each had a history of statin use and hypothyroidism, respectively. In 37 patients, an IVCT was performed (31 patients with MH; six patients with RM); this was positive in all MH patients but only in two of six patients with RM. Eleven patients had fixed muscle weakness (22%; three with MH; eight with RM) (Supplemental table 1). This included mild proximal, predominantly hip girdle weakness (MRC ≥ 4; *n* = 7), ptosis (*n* = 3), facial weakness (*n* = 2) and/or scapular winging (*n* = 1). The two patients with mild facial weakness had remarkable muscle hypertrophy. Two patients had a combination: ptosis or facial weakness with mild proximal weakness. None of the patients had considered those as significant disease symptoms. One patient developed paraparesis following spinal cord injury.

**Table 1:** Summary of clinical features and results of histological analysis

	MH and RM	MH	RM
Clinical features	*n* = 50 (%)	*n* = 31 (%)	*n* = 19 (%)
Sex: male	37 (74)	20 (65)	17 (89)
Positive IVCT (MHS or MHE) or diagnostic MH mutation (according to EMHG)	35 (70)	31 (100)	4 (21)
Permanent muscle weakness	11(22)	3 (10)	8 (42)
Histopathological analysis	*n* = 50	*n* = 31	*n* = 19
Mean age at biopsy (range)	34 (5–67)	37 (10–67)	31 (5–61)
Normal	5 (10)	3 (10)	2 (11)
General myopathic features	45 (90)	28 (90)	17 (89)
Increased fibre size variability	44 (88)	27 (87)	17 (89)
Increased number of fibres with internal nuclei (> 3%)	30 (60)	20 (65)	10 (53)
Increased number of fibres with central nuclei (part of group above)	18 (36)	13 (42)	5 (26)
Type 1 fibre predominance	13 (26)	7 (23)	6 (32)
Prominent lipid vacuoles^(1)^	3 (6)	0 (0)	3 (16)
Necrotic fibres^(2)^	3 (6)	0 (0)	3 (3)
Mild increase in connective tissue in endomysium	2 (4)	1 (3)	1 (5)
Abnormalities on oxidative staining	26 (52)	18 (58)	8 (42)
Unevenness^(3)^	24 (48)	20 (65)	4 (21)
Central cores	7 (14)	6 (19)	1 (5)
Ring -fibres	4 (8)	2 (6)	2 (11)
Multiple minicores	3 (6)	2 (6)	1 (5)
Abnormalities on trichome staining			
Nemaline rods	1 (2)	1 (3)	0 (0)
Electron microscopy (EM)	*n* = 26	*n* = 16	*n* = 10
Normal	4 (15)	3 (19)	1 (10)
Abnormal	22 (85)	13 (81)	9 (90)
Z-band streaming	8 (31)	6 (38)	2 (20)
Cores (central cores or minicores)	8 (31)	6 (38)	2 (20)
Abnormal mitochondria	4 (15)	1 (6)	3 (30)
Lipid droplets	1 (4)	0 (0)	1 (10)
Combined analysis of LM an and EM^a^	*n* = 50	*n* = 31	*n* = 19
Cores (central cores or minicores)	14 (28)	11 (35)	3 (16)
Central cores^a^	8 (16)	7 (23)	1 (5)
Multiple minicores^b^	6 (12)	4 (13)	2 (11)

^a^Central cores were observed by light microscopy (LM) in patients 3, 4, 11, 19, 20, 24 (all MH) and 35 (rhabdomyolysis); and on EM in patients 19, 24, 29 (all MH), and 35 (rhabdomyolysis)

^b^Multiple minicores were observed by LM in patients 21 and 27 (MH) and 47 (rhabdomyolysis), and on EM in patients 5, 27, 31 (MH), 44 and 47 (rhabdomyolysis)

^(1)^ and ^(2)^*p* = 0.02 (Chi-square test)

^(3)^*p* < 0.05 (Chi-square test)

### Molecular genetic studies

The results of the genetic analysis are shown in Table 2: In 30 patients, a diagnostic MH mutation as defined by the EMHG was detected (26 of 30 with MH, and in 4 of 18 with RM). Recurrent mutations were: c.1021G > A (p.Gly341Arg) (*n* = 5 MH), c.1840C > T (p.Arg614Cys) (*n* = 4 MH), c.12861_12869dup (p.Thr4288_Ala4290dup) (*n* = 3 RM), c.7025A > G (p.Asn2342Ser) (*n* = 1 MH;*n* = 1 RM), c.7300G > A (p.Gly2434Arg) (*n* = 5; 2 MH and 3 RM), and c.14545G > A (p.Val4849Ile) (*n* = 10; 9 MH and 1 RM in combination with a second mutation). In addition, two patients had an allele with three *RYR1* mutations previously described [28]. In total, 30 patients had one or two pathogenic mutation(s) (26 with MH; 4 with RM), in three patients the mutation(s) was likely pathogenic (three with RM), and in 17 the mutation(s) were classified as a VUS (five with MH; 12 with RM). Supplemental file 1 shows the additional genetic tests that were performed and showed no other genetic cause for the RM.

**Table 2:** Summary of genetic analysis

Mutation	Number of patients	Number of patients with MH	Number of patients with RM	(Allele with) diagnostic MH mutation	Classification of pathogenicity for MH (or for congenital myopathy in case of ^a^)	Previous report of this case First report; functional characterization
c.1021G > A (p.Gly341Arg)	5	5		Yes	Pathogenic	Quane [29]; Tong [30]
c.1522G > C (p.Glu508Gln)	1		1	No	VUS	This report
c.1597C > T (p.Arg533Cys)	1		1	No	Likely pathogenic	Tammaro [31]; Sato [32]
c.1840C > T (p.Arg614Cys)	4	4		Yes	Pathogenic	Gillard [33]; Girard [34]
c.1840C > T (p.Arg614Cys) c.14364 + 1G > T (on two alleles)	1	1		Yes No	Pathogenic Pathogenic	Gillard [33] for first mutation Girard [34]/Snoeck [21] for second mutation
c.1840C > T (p.Arg614Cys) c. 8026C > T (p.Arg2676Trp) (on two alleles)	1	1		Yes No	Pathogenic Likely pathogenic	Gillard [33]; Girard [34] for first mutation Guis (2004) [35] for second mutation
c.2488C > T (p.Arg830Trp) c.10219G > A (p.Ala3407Thr) (on two alleles)	1		1	No No	VUS VUS	Snoeck [21] Molenaar [36]
c.4178A > G (p.Lys1393Arg) c.14210G > A (p.Arg4737Gln) (on two alleles)	1		1	No	VUS Likely pathogenic	Dlamini [5] Monnier [37]; Gomez [38]
c.4711A > G (p.Ile1571Val) c.10097G > A (p.Arg3366His) c.11798A > G (p.Tyr3933Cys) (on one allele)	2	2		No	VUS VUS VUS	Tammaro [39]; Kraeva [28] Duarte [40]; Kraeva [28] Gillies [41]; Kraeva [28]
c.6385G > A (p.Asp2129Asn)	1		1	No	VUS	Dlamini [5]
c.6502G > A (p.Val2168Met)	1	1		Yes	Pathogenic	Manning [42]; Girard [34]
c.6617C > T (p.Thr2206Met)	1	1		Yes	Pathogenic	Manning [42]; Murayama [43]
c.6838G > A (p.Val2280Ile)	1		1	No	Likely benign	Galli [44]
c.7025A > G (p.Asn2342Ser)	2	1	1	No	VUS^a^	Marchant [45]; Zullo [46]
c.7277A > G (p.Tyr2426Cys)	1		1	No	VUS	Dlamini [5]
c.7300G > A (p.Gly2434Arg)	5	2	3	Yes	Pathogenic	Keating [47]; Girard [34]
c.7304G > A (p.Arg2435His)	1	1		Yes	Pathogenic	Zhang [48]; Avila [49]
c.7361G > A (p.Arg2454His)	1	1		Yes	Pathogenic	Barone [50]; Murayama [43]
c.8054C > T (p.Ser2685Phe)	1		1	No	VUS	Scalco [51]
c.8327C > T (p.Ser2776Phe)	1		1	No	Likely benign	This report
c.10219G > T (p.Ala3407Ser)	1		1	No	VUS	Molenaar [36]
c.10616G > A (p.Arg3539His)	1	1		No	VUS^a^	Dekomien [52]
c.10681G > A (p.Gly3561Arg)	1		1	No	Likely pathogenic	This report
c.12861_12869dup p.Thr4288_Ala4290dup	3		3	No	VUS	Levano [53]
c.14545G > A (p.Val4849Ile)	9	9		Yes	Pathogenic	Jungbluth [6]; Merritt [54]
c.14545G > A (p.Val4849Ile) c.6961A > G (p.Ile2321Val)	1		1	Yes No	Pathogenic VUS^a^	Jungbluth [55]; Merritt [54] Robinson [56]
c.14569T > C (p.Phe4857Leu)	1	1		No	VUS	This report
Total number of patients	50	31	19			

^a^Reported as VUS in a autosomal recessive mode of inheritance

In all but one patient the (presumed) mode of inheritance was AD. Patient 34, who suffered from a severe episode of heat- and exercise-induced rhabdomyolysis with severe encephalomyelopathy, resulting in a permanent spinal cord lesion, had two *RYR1* variants on separate alleles [c.2488C > T (p.Arg830Trp),c.10219G > A (p.Ala3407Thr)]. His parents, both carrier of one of the alleles, had no neuromuscular symptoms and normal CK. Supplemental table 1 shows the results of genetic testing of the individual patients.

### Histopathological studies

A total of 53 muscle biopsies (from 50 patients) were reviewed; in three patients two subsequent biopsies had been performed, at an interval of 4, 5 and 15 years, respectively. Ten biopsies were performed by needle (MH: *n* = 1; RM: *n* = 9), and 43 by open biopsy (MH: *n* = 31; RM: *n* = 3). Mean age at biopsy was 34 years (range 5–67 years).

**Fig. 1:**
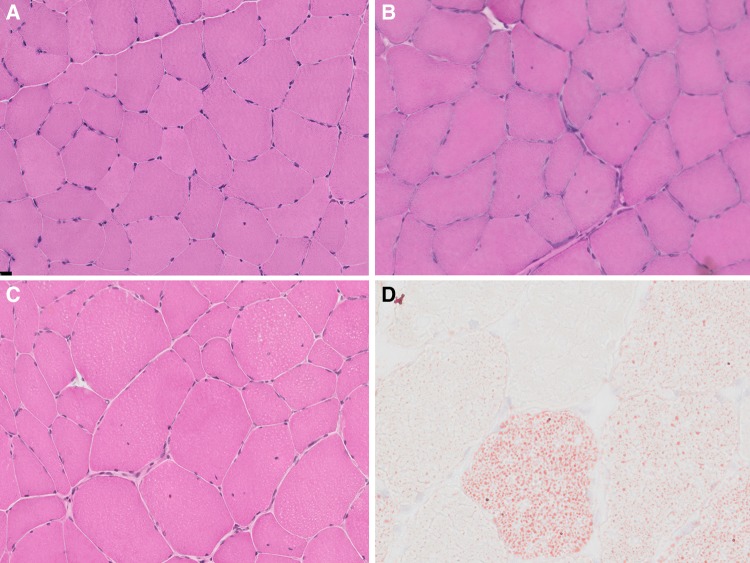
Histopathological features—haematoxylin & eosin (H&E) and Oil red O stain: features on H&E (**a**–**c**) range from normal appearance (**a** Patient 7) to increased fibre size variability and an increase in internal nuclei (**b** Patient 6, and **c** Patient 4), many of them central (**b**). On Oil red O stain (**d** Patient 34), there is marked increase in intracellular lipid, probably related to the timing of the muscle biopsy in relation to the RM episode in the patient

**Fig. 2:**
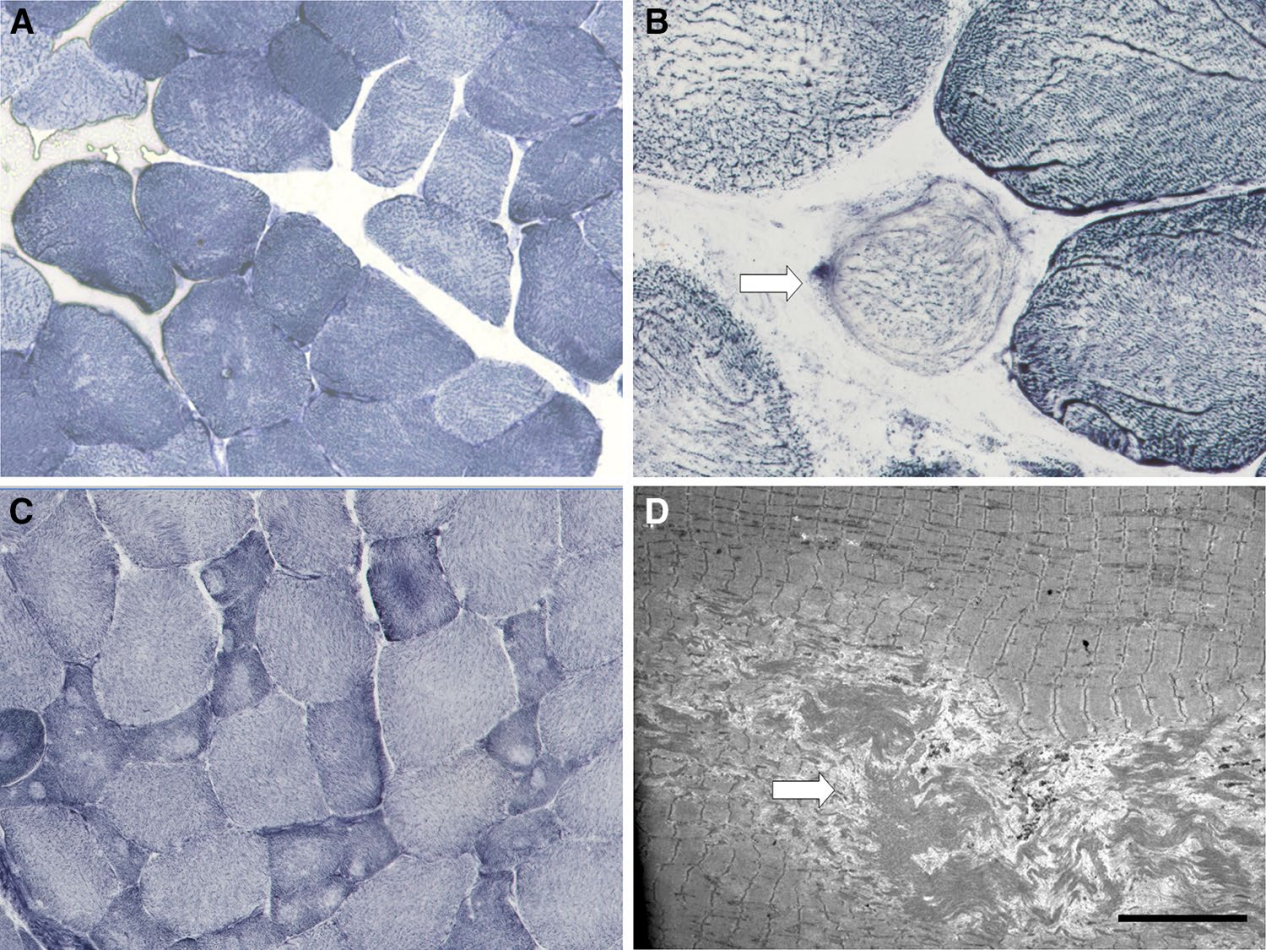
Histopathological features—oxidative stains and electron microscopy: abnormalities on oxidative stains range from mild unevenness of staining (**a** Patient 7) to overt cores and fibre type disproportion on NADH-TR stain (**c** Patient 4). Ring fibres (arrow) (**b** Patient 15) were noted in some cases. On electron microscopy, unstructured central cores (arrow) (**d** Patient 35) were occasionally noted. Size bar = 10 µm

Overall, 90% of the patients showed one or more of the following myopathic features, considered to be definitely abnormal but non-specific: increase in the number of fibres with internal nuclei *n* = 30 of 50 patients (60%) which were centralized in 18 patients (36%), increased fibre size variation (*n* = 44; 88%), and type I (≥ 55%) fibre predominance (*n* = 13; 26%) (Fig. 1b, c). Qualitative evaluation of the biopsies showed fibre type disproportion in patient 4, 28, 43, and 44 (for example: patient 4 in Fig. 2c). Furthermore, ring fibres were seen in four patients (8%; patient 15, 16, 28 with MH and 45 with RM; Fig. 2b), and prominent intracellular lipid-droplets were observed in three patients with RM (patient 34, 36, 46; Fig. 1d). In two of them (patient 34, 46) the biopsy was taken within the first 2 weeks after the RM episode and the finding was considered secondary to propofol infusion on the Intensive Care Unit (ICU). Mild increase in endomysial connective tissue was observed in two patients (patient 20 with MH, 49 with RM). Five patients (10%) had a normal biopsy (Fig. 1a), including four with MH (all with a pathogenic mutation) and one RM case (with a VUS).

Additional abnormalities on oxidative staining (generally considered to be more specific for *RYR1*-related congenital myopathies) were observed in 52% of cases, and included unevenness (or ‘moth-eaten appearance’) (*n* = 24; 48%), central cores (*n* = 7; 14%), or multiple minicores (*n* = 3; 6%). In one patient the muscle biopsy showed features compatible with a rod-core myopathy (patient 29). The differences between the MH and RM groups were non-significant, except for the occurrence of unevenness on oxidative staining (more prevalent in MH; *p* < 0.01), and the presence of intracellular lipid droplets (*p* = 0.02) and necrotic fibres (*p* = 0.02), more prevalent in patients with RM. Overall, the presence of cores neither correlated with muscle weakness nor with presence of (likely) pathogenic mutations. Furthermore, the cores were mostly located in the type 1 fibres and were almost all unstructured on electron microscopy. There was a high prevalence of cores in patients who carried the c.1840C > T mutation located at the *RYR1* N-terminus and classified as a diagnostic MH mutation by the EMHG (three of six patients). Taken together, there was a wide spectrum of histopathological abnormalities, ranging from mild increase in internal nuclei, fibre size variation and unevenness of oxidative staining to more overt core histopathology.

EM analysis could be performed in 26 patients and was abnormal in 22 (85%); all of them had shown abnormalities on light microscopy. Ultrastructural abnormalities included Z-band streaming (*n* = 8; 31%), cores of varying size (*n* = 8; 31%), altered mitochondria (e.g., enlargement, paracrystalline inclusions *n* = 4; 15%), and increased lipid droplets (*n* = 1; 4%). The combined results of LM and EM showed cores in 28% of patients.

In three patients patient 10 (4 years interval), 38 (15 years interval) and 47 (5 years interval) sequential biopsies had been performed (both from the vastus lateralis). Biopsies in patient 10 (MH) showed progression of myopathic features (increase of mild fibre-diameter variation and of internal nuclei), and emergence of oxidative staining abnormalities (mini-core-like morphology) over time. In patient 38 (RM and MH) no significant changes were seen between the two biopsies (performed at age 41 and 59). The second biopsy (performed at age 18, 5 years later) in patient 47 was not available for review but evidence of multi-minicores was noted in the pathology report. Overall, the patients found to have cores on histopathological analysis tended to be slightly older than those without (mean age 37 versus 33 years, *p* > 0.05).

Morphology varied among individuals with the same mutation, and there was no obvious genotype-phenotype correlation at the histopathological level. The most remarkable difference was observed between patients 32 and 33 (mother and daughter with RM, with *RYR1* p.G2434R; age at vastus lateralis biopsy 45 and 15 years, respectively) (Supplementary File 2). This might be related to the progression of morphological changes over time and/or variability of histopathological features between individuals with the same genetic background.

## Discussion

The present retrospective multicentre study includes a total of 50 cases with *RYR1*-related MH and/or RM, and represents the largest series reported to date describing the histopathological spectrum associated with these conditions. We found a broad spectrum of histopathological features, ranging from a normal muscle biopsy appearance (10% of patients, MH: *n* = 4; RM: *n* = 1) to overtly myopathic changes, including those considered to be non-specific (in particular, and in order of decreasing frequency, an increase in fibre size variability, increase of internal nuclei, and type 1 fibre predominance), and, less frequently, those considered to be more closely associated with *RYR1*-related myopathies (in particular, and in order of decreasing frequency, unevenness of oxidative stains, and more distinct central core and minicore structures, as well as, rarely, the combination of cores and rods).

These findings suggest that, whilst clearly leaning towards the milder end of the spectrum, *RYR1*-related MH and RM nevertheless form part of a histopathological continuum with the better-characterized *RYR1*-related myopathies, including CCD, MmD, CNM, and CFTD. Along the same lines, it is arguable that even some of the histopathological features considered to be non-specific, increase of internal or even central nuclei, increased fibre size variability, and type 1 fibre predominance may represent minor manifestations of well-recognized *RYR1*-related congenital myopathies [3, 4, 57]. Of note, similar mild myopathic manifestations have been previously reported in two other phenotypes closely linked to MH-associated *RYR1* mutations, the King-Denborough syndrome and late-onset axial myopathy [9, 58]. Remarkably, histopathological features in *RYR1*-related MH and RM were not distinguishable, supporting our previous notion [5] that these are different but related manifestations due to genetically and functionally similar dominant *RYR1* gain-of-function mutations.

Other histopathological features not previously highlighted in *RYR1*-related disorders included the presence of sporadic ring fibres seen in four patients, two with MH and RM each. The significance of ring fibres, previously reported in MH, is not known, although some have considered them an indicator of degeneration or regeneration [59]. Features of muscle degeneration and regeneration, however, were conspicuously absent in the cases studied, although developmental and fetal myosins were not studied. There was also no increase in fat and connective tissue, a feature which is common in severe early-onset *RYR1*-related myopathies that may occasionally mimic a congenital muscular dystrophy, and may also relate to the part of the quadriceps sampled because of differential muscle involvement. Intracellular lipid droplets have not previously been reported in association with *RYR1* mutations and were observed in three patients presenting with RM. Progression of muscle pathology, which has been noted in *RYR1*-related myopathies but also in one MH pedigree where muscle biopsies were performed at different ages [10, 19], was observed in two of the three patients in whom subsequent biopsies had been performed.

Our findings also support the earlier observation that there is no direct correlation between muscle pathology (in particular, the presence of cores) and muscle weakness in *RYR1*-related myopathies: only one patient with mild muscle weakness had central cores, whereas five patients with mild proximal muscle weakness showed no core pathology at all (Supplementary File 1 and 2). Cores (both central and multiple minicores) were seen in 50% of patients with the *RYR1* N-terminal MH mutation c.1840C > T; it is uncertain if this reflects a specific genotype-phenotype correlation or is merely a co-incidental finding related to the relative high frequency of this causative MH mutation. The presence of mild weakness in patients originally referred for the investigation of MH suggests that some of these patients may in fact have had a mild *RYR1*-related myopathy, in cases where cores were present even fulfilling the criteria for either Central Core Disease (CCD) or Multi-minicore Disease (MmD); this is an important observation emphasizing that MH patients could benefit from a formal assessment by a neurologist with experience in neuromuscular disorders, something which is currently not performed as a standard at all MH centres.

Findings from our systematic muscle biopsy review focussing on the histopathological features of *RYR1*-related MH and RM are supported by the few studies primarily focussing on clinical and genetic aspects of these presentations where such features have also been occasionally reported. The first larger case series on *RYR1*-related RM suggested similar non-specific but unequivocal changes, comprising increased variability in fibre size, increased internal nucleation and unevenness or core-like structures on oxidative stains in the few cases where this was studied [5, 15]. Rueffert et al. reported histopathological changes in eight members of a large MH family carrying the *RYR1* c.6617C > T (p.Thr2206Met) mutation, all of them showing mild myopathic changes with isolated hypotrophic fibres and disseminated small areas with the reduction of oxidative staining (“multi-minicore like” lesions) [19]. In contrast, Orlov showed histopathological changes in only 28 of 86 (33%) of genetically-confirmed MH patients [22]. Our recent retrospective study of 77 unrelated patients with *RYR1*-related disorders also included some histopathological data based on a review of 18 muscle biopsy reports (but not the original muscle biopsy slides) from patients with MH or RM, suggesting increase in internal nuclei (13/18, 72%), unevenness of oxidative staining (10/18, 56%) and increased fibre size variation in (8/18, 44%) as the most common histopathological features [21]. Fibre type I predominance, central cores, or multi-minicores were not reported, emphasizing the merit of our more extensive study exclusively focussing on *RYR1*-related MH and RM in a much larger cohort, and, most importantly, based on a systematic review of all muscle biopsies by the same neuromuscular pathologists.

Although the main emphasis of the present study was on the histopathological features of *RYR1*-related MH and RM, we gained some additional important insights, in particular concerning the relationship between clinical phenotypes, histopathological appearances, outcome of the IVCT, and genotype. The three MH patients with a positive IVCT had a normal muscle biopsy, whereas three patients with RM and a negative IVCT had an abnormal muscle biopsy with histopathological features suggestive of *RYR1* involvement. These findings are in line with previous reports showing absence of a correlation between the IVCT result and the presence of histopathological changes [21, 60, 61], and, more importantly, emphasize that at least in *RYR1*-related RM, a diagnostic muscle biopsy may be as important as the IVCT for a comprehensive pathogenicity assessment of any *RYR1* variant of uncertain significance identified. Only four patients with *RYR1*-related RM carry a diagnostic *RYR1* mutation according to the EMGH criteria, and the IVCT was positive in only two of the six patients tested, one of which was later retrospectively found to have a diagnostic MH mutation. Hence, in contrast to inconclusive genetic and normal IVCT results, a suggestive combination of histopathological features was more frequently indicative of *RYR1* involvement than the IVCT result.

Based on the observations outlined above, in cases where *RYR1*-related RM is suspected [51], we propose the following diagnostic steps: (1) *RYR1* Sanger sequencing (or preferably panel or exome sequencing to exclude other genetic causes of RM); (2) in case of a diagnostic MH mutation [i.e., a functionally characterized *RYR1* mutation recognized as MH causative, as documented on the EMHG website (http://www.emhg.org)], or an unequivocally pathogenic variant in another known RM-associated gene, no further tests need to be performed; where a diagnostic MH mutation is identified, the patient and his or her familiy needs to be counselled accordingly; (3) in case of a *RYR1* variant which has been associated with MH in the past but is not a diagnostic MH mutation as defined above, perform an IVCT to determine the MH risk with a functional study to prove causality as next step, and perform histopathological investigation; (4) in case of a *RYR1* variant not previously associated with MH, perform a diagnostic needle biopsy to evaluate the histopathological changes.

These diagnostic guidelines based on expert opinion emphasize the need for future research, in particular aimed at the large-scale functional characterization of *RYR1* variants implicated in RM, and the importance of international collaborative mutational databases to establish clinico-pathological genotype-phenotype correlations for RM and other *RYR1*-related disorders in larger cohorts, ultimately reducing the need for invasive investigations such as a muscle biopsy [62].

The main limitations of this study are its partly retrospective design, resulting in missing data for several patients and only qualitative data for certain features, and the fact that the pathologists reviewing the muscle biopsies were not blinded to the genetic diagnosis. Another limitation is the absence of data on age-matched control subjects, largely reflecting the fact that data on muscle histology in healthy controls are limited. We recently included 12 healthy subjects as controls in a study on inclusion body myositis and observed mild myopathic features (increase of fibre size variation and of internal nuclei) in at least half of them. However, the subjects were older than the patients in this current study (mean age 54 years versus 34 years), which is likely to be relevant considering that age-related muscle decline has been recognized to start only from the fifth decade (Lassche, personal communication) [63]. Despite these shortcomings, we do think that the results of the present study offer valid preliminary and robust insights into the histopathological spectrum of MH and RM. With regards to future work, the diagnostic value of the muscle biopsies in this context should preferably be tested systematically in a prospective study with blinded pathology reviewers and well-defined quantitative criteria, and, if feasible, biopsies from age-matched control subjects.

In conclusion, our findings illustrate that *RYR1*-related MH and RM show a very similar histopathological spectrum, ranging from non-specific findings to features considered to be more suggestive of *RYR1*-related congenital myopathies such as central nuclei, central cores, multiple mini-cores and unevenness of oxidative staining. Although many of those features are often considered non-specific when occurring in isolation, their common occurrence should raise the suspicion of a *RYR1*-related disorder, and/or support the likelihood of causality where *RYR1* variants of only uncertain pathogenicity have been identified in a patient. Until more robust methods of functional characterization and/or large-scale genotype-phenotype data will become available, (needle) muscle biopsy will remain an additional but essential tool in the ascertainment of presumed *RYR1*-related RM.

## Electronic supplementary material

Below is the link to the electronic supplementary material.

Supplementary material 1 Supplementary files We identified 50 patients from 46 families, including 31 MH cases (16 index patients and 15 first-degree adult relatives carrying the familial RYR1 variant who volunteered to undergo the IVCT instead of the mostly paediatric index patient) and 19 individuals who had suffered from RM. One patient (Patient 38) within the RM group had an initial episode of exertional RM followed by an MH event. Four pairs of related patients were included (patients 6 and 7, 15 and 16, 23 and 24 in the MH group, and 32 and 33 in the RM group). Supplementary File 1: Clinical data of individual patients. Excel sheet 1 shows sex, clinical diagnosis, *RYR1* mutation(s) and exon, the classification of pathogenecity and presumed inheritance, the absence or presence of an allele with a diagnostic mutation for MH (according to EMHG list), the IVCT result, the presence of fixed muscle weakness, and if available, previous report(s) of the cases. Column O shows the other genetic tests performed in patients with RM, including next generation sequencing or Sanger sequencing of genes involved in metabolic and pseudometabolic myopathies in most patients. (DOCX 36 KB)

Supplementary material 2 Supplementary File 2: Histological data of individual patients. Excel sheet 2 shows the pathogenic or likely pathogenic mutation, age at biopsy, type of biopsy, comments on general morphology, fiber variation, internal nuclei (> 3%; central or internalized), the presence of basophilic fibers, fiber type predominance (T1 ≥ 55%) (ATP 4,2), variation T1 and T2%, abnormalities in the oxidative staining, presence of COX negative fibers or rods (nemaline rods), or fat droplets, and results of electron microscopy (EM). I: internal nuclei; c: central nuclei (DOCX 48 KB)
