# Supplementary material for: The histopathological spectrum of malignant hyperthermia and rhabdomyolysis due to RYR1 mutations
Source: J Neurol. 2019 Feb 20;266(4):876–87. doi: 10.1007/s00415-019-09209-z (PMC6420893; doi:10.1007/s00415-019-09209-z)
Supplement: Supplementary file 1 — Supplementary material 1 Supplementary files We identified 50 patients from 46 families, including 31 MH cases (16 index patients and 15 first-degree adult relatives carrying the familial RYR1 variant who volunteered to undergo the IVCT instead of the mostly paediatric index patient) and 19 individuals who had suffered from RM. One patient (Patient 38) within the RM group had an initial episode of exertional RM followed by an MH event. Four pairs of related patients were included (patients 6 and 7, 15 and 16, 23 and 24 in the MH group, and 32 and 33 in the RM group). Supplementary File 1: Clinical data of individual patients. Excel sheet 1 shows sex, clinical diagnosis, RYR1 mutation(s) and exon, the classification of pathogenecity and presumed inheritance, the absence or presence of an allele with a diagnostic mutation for MH (according to EMHG list), the IVCT result, the presence of fixed muscle weakness, and if available, previous report(s) of the cases. Column O shows the other genetic tests performed in patients with RM, including next generation sequencing or Sanger sequencing of genes involved in metabolic and pseudometabolic myopathies in most patients. (DOCX 36 KB) [file 415_2019_9209_MOESM1_ESM.docx]

| Study # | Sex | Diagnosis  (for RM n = number of RM episodes) | *RYR1* mutation(s) | Exon | Classification of pathogenecity | Presumed inheritance | Allelle with diagnostic mutation for MH (according to EMHG list) | IVCT result | Fixed muscle weakness | Previous report of this case | |  | Other genetic tests performed |
| --- | --- | --- | --- | --- | --- | --- | --- | --- | --- | --- | --- | --- | --- |
| ***MALIGNANT HYPERTHERMIA*** | | | | | | | | | | | | | |
| **1** | M | MH in son | c.14545G>A (p.Val4849Ile) | 101 | pathogenic | AD | yes | MHS | no | Snoeck 2015 |  |  |  |
| **2** | F | MH | c.14545G>A (p.Val4849Ile) | 101 | pathogenic | AD | yes | MHS | no | Snoeck 2015 |  |  |  |
| **3** | F | MH | c.1840C>T (p.Arg614Cys) | 17 | pathogenic | AD | yes | MHS | no | Snoeck 2015 |  |  |  |
|  |  |  | c.14364+1G>T | 99 | pathogenic |  |  |  |  |  |  |  |  |
| **4** | M | MH | c.10616G>A (p.(Arg3539His) (brother) | 71 | VUS / likely benign | AD | no | MHS | no | Snoeck 2015 |  |  |  |
| **5** | M | MH | c. 1840C>T (p.(Arg614Cys)) | 17 | pathogenic | AD | yes | MHE | no | Snoeck 2015 |  |  |  |
|  |  |  | c. 8026C>T (p.Arg2676Trp) | 50 | likely pathogenic |  |  |  |  |  |  |  |  |
| **6 (F of 7)** | M | MH | c.14545G>A (p.Val4849Ile) | 101 | pathogenic | AD | yes | MHS | no | Snoeck 2015 |  |  |  |
| **7 (S of 6)** | F | MH in brother | c.14545G>A (p.Val4849Ile) | 101 | pathogenic | AD | yes | MHS | no | Snoeck 2015 |  |  |  |
| **8** | F | MH | c.14545G>A (p.Val4849Ile) | 101 | pathogenic | AD | yes | MHS | mild proximal weakness in legs | Snoeck 2015 |  |  |  |
| **9** | M | MH in son | c.14545G>A (p.Val4849Ile) | 101 | pathogenic | AD | yes | MHS | no | Snoeck 2015 |  |  |  |
| **10** | F | MH in son, exertional myalgia and cramps | c.14545G>A (p.Val4849Ile) | 101 | pathogenic | AD | yes | MHS | mild proximal weakness in legs | Snoeck 2015 |  |  |  |
| **11** | F | MH | c.1840C>T (p.Arg614Cys) | 17 | pathogenic | AD | yes | MHS | no | Snoeck 2015 |  |  |  |
| **12** | M | MH | c.1840C>T (p.Arg614Cys) | 17 | pathogenic | AD | yes | MHS | no | Snoeck 2015 |  |  |  |
| **13** | M | MH | c.7025A>G (p.Asn2342Ser) | 43 | VUS / likely benign | AD | no | MHE | no | Snoeck 2015 |  |  |  |
| **14** | F | MH | c.7361G>A (p.Arg2454His) | 46 | pathogenic | AD | yes | MHS | no | Snoeck 2015 |  |  |  |
| **15 (F of 16)** | M | MH in son of brother | c.7300G>A (p.Gly2434Arg) | 45 | pathogenic | AD | yes | MHS | no | Snoeck 2015 |  |  |  |
| **16 (F of 15)** | M | MH in son | c.7300G>A (p.Gly2434Arg) | 45 | pathogenic | AD | yes | MHS | no | Snoeck 2015 |  |  |  |
| **17** | M | MH in son | c.14545G>A (p.Val4849Ile) | 101 | pathogenic | AD | yes | MHS | no |  |  |  |  |
| **18** | M | MH in maternal nephew | c.1021G>A (p.Gly341Arg) | 11 | pathogenic | AD | yes | MHS | no |  |  |  |  |
| **19** | M | MH | c.14569T>C (p.Phe4857Leu) | 101 | VUS | AD | no | MHS | Mild proximal weakness |  |  |  |  |
| **20** | F | MH in child | c.1021G>A (p.Gly341Arg) | 11 | pathogenic | AD | yes | MHS | no |  |  |  |  |
| **21** | M | MH in sib | c.6502G>A (p.Val2168Met) | 39 | pathogenic | AD | yes | MHS | no |  |  |  |  |
| **22** | M | MH | c.1021G>A (p.Gly341Arg) | 11 | pathogenic | AD | yes | MHS | no |  |  |  |  |
| **23 (D of 24)** | F | MH in brother | c.4711A>G (p.Ile1571Val) | 33 | VUS | AD | no | MHS | no | Kraeva 2015 |  |  |  |
|  |  |  | c.10097G>A (p.Arg3366His) | 67 | VUS |  |  |  |  |  |  |  |  |
|  |  |  | c.11798A>G (p.Tyr3933Cys) | 86 | VUS |  |  |  |  |  |  |  |  |
| **24 (F of 23)** | M | MH in son | c.14545G>A (p.Val4849Ile) | 101 | pathogenic | AD | yes | MHS | no |  |  |  |  |
| **25** | M | MH | c.4711A>G (p.Ile1571Val) | 33 | VUS | AD | no | MHS | no | Kraeva 2015 |  |  |  |
|  |  |  | c.10097G>A (p.Arg3366His) | 67 | VUS |  |  |  |  |  |  |  |  |
|  |  |  | c.11798A>G (p.Tyr3933Cys) | 86 | VUS |  |  |  |  |  |  |  |  |
| **26** | M | MH | c.1021G>A (p.Gly341Arg) | 11 | pathogenic | AD | yes | MHS | no | Dhaese 2010 |  |  |  |
| **27** | M | MH | c.7304G>A (p.Arg2435His) | 45 | pathogenic | AD | yes | MHS | no |  |  |  |  |
| **28** | F | MH | c.1840C>T (p.Arg614Cys) | 17 | pathogenic | AD | yes | MHS | no |  |  |  |  |
| **29** | F | MH in aunt | c.1840C>T (p.Arg614Cys) | 17 | pathogenic | AD | yes | MHS | no |  |  |  |  |
| **30** | M | MH in sib | c.1021G>A (p.Gly341Arg) | 11 | pathogenic | AD | yes | MHS | no |  |  |  |  |
| **31** | M | MH in child | c.6617C>T (p.Thr2206Arg) | 40 | pathogenic | AD | yes | MHS | no |  |  |  |  |

| ***RHABDOMYOLYSIS*** | | | | | | | | | | | | | |
| --- | --- | --- | --- | --- | --- | --- | --- | --- | --- | --- | --- | --- | --- |
| **32 (M of 33)** | F | Recurrent exertional rhabdomyolysis (n=2) | c.7300G>A (p.Gly2434Arg) | 45 | pathogenic | AD | yes | Not performed, MHS | Mild proximal weakness | Dlamini 2013 | Snoeck 2015 |  | Lysosomal enzyme activity (Pompe's disease); metabolic screening, mtDNA |
| **33 (child of 32)** | F | Recurrent exertional rhabdomyolysis (n=8) | c.7300G>A (p.Gly2434Arg) | 45 | pathogenic | AD | yes | Not performed, MHS | Mild ptosis | Dlamini 2013 | Snoeck 2015 | Scalco 2016 | Lysosomal enzyme activity (Pompe's disease); metabolic screening, mtDNA |
| **34** | M | Heat- and exercise-induced rhabdomyolysis with severe encephalomyelopathy (n=1) | c.2488C>T (p.Arg830Trp) | 20 | VUS | AR | no | MHN | Spinal cord lesion | Snoeck 2015 |  |  | NGS for myopathies and NGS open exome |
|  |  |  | c.10219G>A (p.Ala3407Thr) | 67 | VUS |  |  |  |  |  |  |  |  |
| **35** | M | Exertional rhabdomyolysis induced by hypothyroidism  (n=2) | c.6385G>A (p.Asp2129Asn) | 39 | VUS | AD | no | Not performed | no | Dlamini 2013 | Snoeck 2015 |  | No additional tests |
| **36** | M | Recurrent exertional rhabdomyolysis (n=4) | c.14545G>A (p.Val4849Ile) | 101 | pathogenic | AD | yes | MHS | Mild ptosis and mild scapular winging | Snoeck 2016 |  |  | Metabolic screening, CPT2, PYGM |
|  |  |  | c.6961A>G (p.Ile2321Val) | 43 | VUS / likely benign |  |  |  |  |  |  |  |  |
| **37** | M | Exertional rhabdomyolysis (n=1) | c.10681G>A (p.Gly3561Arg) | 71 | likely pathogenic | AD | no | MHN | no | Dlamini 2013 | Snoeck 2015 |  | Metabolic screening, mitochondrial enzym activity |
| **38** | M | Exertional rhabdomyolysis (CK 4800 U/l) and MH (CK 2391 U/l)* (n=1) | c.4178A>G (p.Lys1393Arg) | 29 | likely benign | AD | no | MHS | no | Dlamini 2013 | Snoeck 2015 |  | No additional tests |
|  |  |  | c.14210G>A (p.Arg4737Gln) | 98 | likely pathogenic |  |  |  |  |  |  |  |  |
| **39** | M | Recurrent exertional rhabdomyolysis (n=2) | c.7277A>G (p.Tyr2426Cys) | 45 | VUS | AD | no | MHN | no | Dlamini 2013 | Snoeck 2015 |  | No additional tests |
| **40** | M | Infection-induced rhabdomyolsis and mild axial myopathy (n=2) | c.10219G>T (p.(Ala3407Ser)) | 67 | VUS | AD | no | MHN | Mild proximal weakness in legs | Molenaar 2014 | Snoeck 2015 |  | Metabolic screening, mitochondrial enzym activity, CPT2 |
| **41** | M | Statin-induced rhabdomyolysis (n=2) | c.8327C>T (p.Ser2776Phe) | 53 | likely benign | AD | no | Not performed | no | Snoeck 2015 |  |  | No additional tests |
| **42** | M | Exertional rhabdomyolysis  (n>10) | c.12861_12869dup (p.Thr4288_Ala4290dup) | 91 | VUS | AD | no | Not performed | no | Dlamini 2013 |  |  | Exclusion of common metabolic (in particular, glycogen storage and fatty oxidation) disorders |
| **43** | M | Exertional rhabdomyolysis  (recurrent) | c.12861_12869dup (p.Thr4288_Ala4290dup) | 91 | VUS | AD | no | Not performed | Mild proximal weakness | Dlamini 2013 |  |  | Exclusion of common metabolic (in particular, glycogen storage and fatty oxidation) disorders |
| **44** | M | Heat- and exercise-induced rhabdomyolysis | c.12861_12869dup (p.Thr4288_Ala4290dup) | 91 | VUS | AD | no | Not performed | no | Dlamini 2013 |  |  | Exclusion of common metabolic (in particular, glycogen storage and fatty oxidation) disorders |
| **45** | M | Recurrent exertional myalgia and rhabdomyolysis (n=1) | c.1522G>C (p.(Glu508Gln) | 14 | VUS | AD | no | Not performed | Mild ptosis, mild proximal weakness |  |  |  | NGS for rhabdomyolysis and metabolic myopathies, mtDNA |
| **46** | M | Recurrent exertional rhabdomyolysis | c.1597C>T (p.Arg533Cys) | 15 | likely pathogenic | AD | no | Not performed | Mild facial weakness; marked muscle hypertrophy |  |  |  | NGS for rhabdomyolysis and metabolic myopathies |
| **47** | M | Recurrent exertional rhabdomyolysis | c.8054C>T (p.Ser2685Phe) | 50 | VUS | AD | no | Not performed | No | Scalco 2016 |  |  | NGS channelopathy panel and NGS rhabdomyolysis panel |
| **48** | M | Exertional myalgia and recurrent rhabdomyolysis in son | c.6838G>A (p.Val2280Ile) | 42 | VUS / likely benign | AD | no | Not performed | No | Scalco 2016 |  |  | NGS for rhabdomyolysis and metabolic myopathies, metabolic screening, mitochondrial enzym activity |
| **49** | M | Exertional recurrent rhabdomyolysis and cramps | c.7300G>A (p.Gly2434Arg) | 45 | pathogenic | AD | yes | Not performed | Mild facial weakness; marked muscle hypertrophy |  |  |  | NGS for rhabdomyolysis and metabolic myopathies |
| **50** | M | Exertional recurrent rhabdomyolysis and cramps | c.7025A>G (p.Asn2342Ser) | 43 | VUS / likely benign | AD | no | Not performed | No |  |  |  | CACNA1S, CPT2, ETFA, ETFB, ETFDH, HADHA |
