# Supplementary material for: The histopathological spectrum of malignant hyperthermia and rhabdomyolysis due to RYR1 mutations
Source: J Neurol. 2019 Feb 20;266(4):876–87. doi: 10.1007/s00415-019-09209-z (PMC6420893; doi:10.1007/s00415-019-09209-z)
Supplement: Supplementary file 2 — Supplementary material 2 Supplementary File 2: Histological data of individual patients. Excel sheet 2 shows the pathogenic or likely pathogenic mutation, age at biopsy, type of biopsy, comments on general morphology, fiber variation, internal nuclei (> 3%; central or internalized), the presence of basophilic fibers, fiber type predominance (T1 ≥ 55%) (ATP 4,2), variation T1 and T2%, abnormalities in the oxidative staining, presence of COX negative fibers or rods (nemaline rods), or fat droplets, and results of electron microscopy (EM). I: internal nuclei; c: central nuclei (DOCX 48 KB) [file 415_2019_9209_MOESM2_ESM.docx]

| Study # | Pathogenic or likely pathogenic mutation | Age at biopsy | Cores = 1 | Type of biopsy | General morphology | Fiber variation | Internal nuclei (>3%) | internal nuclei central or internalized | Multiple internal nuclei in a fiber | Basophilic fibers | Fiber type predominance (T1≥55%) (ATP4,2) |  |  |  | Variation T1 and T2 | % | Abnormalities in the oxidative stainings | COX negative fibers | Rods (nemaline rods) | Fat droplets | EM resultaten |  |  |  |
| --- | --- | --- | --- | --- | --- | --- | --- | --- | --- | --- | --- | --- | --- | --- | --- | --- | --- | --- | --- | --- | --- | --- | --- | --- |
|  |  |  |  |  |  | minimal=+, moderate=++ and severe =+++ | 3-10=+, 10-50=++ and <50=+++ |  | 3-10=+, 10-50=++ and <50=+++ |  |  | smallest T1 (µm) | largest T1 (µm) | smallest T2 (µm) | largest T2 (µm) | %T1 |  |  |  |  | Cores, minicores and other alterations | Rods of other | Cores in T1 and/or T2 | Conclusion by pathologist |
| ***MALIGNANT HYPERTHERMIA*** | | | | | | | | | | | | | | | | | | | | | | | | |
| 1 | 1 | 31 | 0 | open | mild variation and moderate increase of internal nuclei | + | + | i | + | 0 | T2 | 30 | 90 | 40 | 110 | 47 | 0 | 0 | 0 | 0 | n.a. | n.a. | n.a. | Aspecific myopathic changes |
| 2 | 1 | 21 | 0 | open | a few damaged hyaline-fiber | 0 | 0 | 0 | 0 | a few damaged hyaline-fiber | T2 | 50 | 75 | 50 | 79 | 35 | unOS | 0 | 0 | 0 | n.a. | n.a. | n.a. | Small abnormalities; not specific |
| 3 | 1 | 19 | 1 | open | moderate variation and increase of internal nuclei | ++ | ++ | mostly c | + | 0 | T1 | 42 | 133 | 67 | 129 | 53 | cores | 0 | 0 | 0 | n.a. | n.a. | T1 | Core myopathie (not classic) |
| 4 | 0 | 30 | 1 | open | moderate variation and increase of internal nuclei, cores | +++ | ++ | i | ++ | 0 | T1 | 19 | 96 | 10 | 146 | 68 | cores | 0 | 0 | 0 | n.a. | n.a. | T1 (few T2) | Central core myopathy, mild fibertype disproportion |
| 5 | 1 | 56 | 0 | open | split-fibers and ragged red fibers | +++ | ++ | i | ++ | + | T2 | 21 | 167 | 10 | 146 | 31 | ragged red/cox negative | 1 | 0 | 0 | Z-band alterations and multiple minicores. | non | T1 | Mini-core myopathy (not classic) |
| 6 (F of 7) | 1 | 18 | 0 | open | mild variation and increase of internal nuclei | + | + | mostly c | ++ | 0 | T1 | 63 | 108 | 88 | 113 | 71 | 0 | 0 | 0 | 0 | n.a. | n.a. | n.a. | Aspecific myopathic changes |
| 7 (S of 6) | 1 | 38 | 0 | open | normal | 0 | 0 | 0 | 0 | 0 | T2 | 46 | 92 | 63 | 88 | 52 | 0 | 0 | 0 | 0 | n.a. | n.a. | n.a. | Normal |
| 8 | 1 | 33 | 0 | open | mild variation and increase of internal nuclei | ++ | ++ | i and c | ++ | 0 | T2 | 38 | 88 | 54 | 88 | 23 | unOS | 0 | 0 | 0 | n.a. | n.a. | n.a. | Aspecific myopathic changes |
| 9 | 1 | 41 | 0 | open | mild variation and increase of internal nuclei | + | + | i and c | + | 0 | T2 | 46 | 75 | 42 | 82 | 30 | 0 | 0 | 0 | 0 | n.a. | n.a. | n.a. | Minimal aspecific changes |
| 10 | 1 | 33 | 0 | needle | normal/very mild variation | 0 | 0 | 0 | 0 | 0 | T2 | 58 | 83 | 54 | 83 | 45 | 0 | 0 | 0 | 0 | n.a. | n.a. | n.a. | Normal |
| *2nd biopsy* | *1* | *37* | *0* | *open* | *mild variation and increase of internal nuclei, some basophilic fibers* | *+* | *+* | *i and c* | *+* | *+* | *T2* | *55* | *90* | *40* | *85* | *39* | *0* | *0* | *0* | *0* | *n.a.* | *n.a.* | *n.a.* | *Small Aspecific myopathic changes* |
| 11 | 1 | 42 | 1 | open | moderate variation and increase of internal nuclei | ++ | + | i and c | 0 | 0 | T2 | 33 | 79 | 17 | 88 | 39 | cores | 0 | no trichroom | n.a. | n.a. | n.a. | n.a. | Core myopathy (not classic type) |
| 12 | 1 | 37 | 0 | open | mild variation and some increase of internal nuclei | ++ | + | i | 0 | 0 | T2 | 30 | 115 | 45 | 120 | 39 | 0 | 0 | no trichroom | n.a. | n.a. | n.a. | n.a. | Normal |
| 13 | 0 | 10 | 0 | open | moderate variation and increase of internal nuclei | ++ | ++ | i and c | ++ | 0 | T2 | 45 | 90 | 50 | 115 | 31 | 0 | 0 | 0 | 0 | n.a. | n.a. | n.a. | Aspecific myopathic changes |
| 14 | 1 | 26 | 0 | open | mild variation and some increase of internal nuclei | + | 0 | 0 | 0 | + | T2 | 30 | 110 | 25 | 110 | 27 | unOS | 0 | 0 | 0 | larger and abnormal mitochondria. Myelin figures, some Z-line streaming. | non | non | Aspecific myopathic changes |
| 15 (F of 16) | 1 | 42 | 0 | open | moderate variation and increase of internal nuclei | ++ | ++ | i and c | ++ | 0 | T2 | 71 | 117 | 79 | 133 | 44 | unOS, one ringfiber | 0 | 0 | 0 | n.a. | n.a. | n.a. | Myopathic changes |
| 16 (F of 15) | 1 | 39 | 0 | open | moderate variation and increase of internal nuclei | ++ | + | i | + | some fragmented fibers | T2 | 34 | 89 | 17 | 82 | 41 | one ringfiber | 0 | 0 | 0 | n.a. | n.a. | n.a. | Aspecific myopathic changes |
| 17 | 1 | 67 | 0 | open | moderate variation and increase of internal nuclei | ++ | ++ | i and c | ++ | 0 | T2 | 33 | 83 | 29 | 92 | 38 | 0 | 0 | 0 | 0 | Z-band streaming | non | non | Aspecific myopathic changes |
| 18 | 1 | 35 | 0 | open | moderate variation | ++ | ++ | i and c | ++ | 1 | T2 | n.a. | n.a. | n.a. | n.a. | T2 normal | central clearing and unOS | n.a. | 0 | 0 | normal | non | non | Aspecific myopathic changes |
| 19 | 0 | 22 | 1 | open | minimal variation and mostly central nuclei | + | ++ | mostly c | + | 0 | T1 | n.a. | n.a. | n.a. | n.a. | T1, 100% | central cores | n.a. | 0 | 0 | cores, mostly structured | non | T1 | Central core myopathy |
| 20 | 1 | 59 | 1 | open | moderate variation, split-fibers and fibrosing | ++ | 0 | 0 | 0 | splitted fibers | T1 | n.a. | n.a. | n.a. | n.a. | T1, 60% | cores in T1, atrofic T1, moth eaten, lobulated fibers | n.a. | 0 | 0 | myogenic changes | non | T1 | (Mini-)core myopathy (not classic) |
| 21 | 1 | 32 | 1 | open | moderate variation and increase of internal nuclei | + | ++ | i | ++ | 0 | T2 | n.a. | n.a. | n.a. | n.a. | T2 normal | moth eaten, multimini core-like | n.a. | 0 | 0 | n.a. | n.a. | n.a. | Aspecific myopathic changes |
| 22 | 1 | 19 | 0 | open | normal | 0 | 0 | 0 | 0 | 0 | T2 | n.a. | n.a. | n.a. | n.a. | T2 normal | 0 | n.a. | 0 | 0 | normal | non | non | Normal |
| 23 (D of 24) | 0 | 35 | 0 | open | mild variation | + | 0 | 0 | 0 | 0 | T2 | n.a. | n.a. | n.a. | n.a. | T2 normal | unOS | n.a. | 0 | 0 | normal | non | non | Small aspecific changes |
| 24 (F of 23) | 1 | 61 | 1 | open | mild variation | + | 0 | 0 | 0 | 0 | T2 | n.a. | n.a. | n.a. | n.a. | T2 normal | central cores | n.a. | 0 | 0 | z-band streaming, central cores | non | T1 | Central core myopathy |
| 25 | 0 | 66 | 0 | open | moderate variation, increase of internal nuclei and nuclarclumbs | ++ | 0 | 0 | 0 | 0 | T2 | n.a. | n.a. | n.a. | n.a. | T2 normal | 0 | n.a. | 0 | 0 | z-band streaming, minimal myofibrial changes | non | non | Aspecific myopathic changes |
| 26 | 1 | 47 | 0 | open | minimal variation and increase of internal nuclei | + | + | i and c | + | 0 | T1 | n.a. | n.a. | n.a. | n.a. | T1, 60% (clumping of T1) | minimal unOS | n.a. | 0 | 0 | myogenic changes, no cores | non | non | Aspecific myopathic changes |
| 27 | 1 | 24 | 1 | open | normal | 0 | 0 | 0 | 0 | 0 | T2 | n.a. | n.a. | n.a. | n.a. | T2 normal | multimini core-like | n.a. | 0 | 0 | minicores and one core-like structure | non | T1 | Suggestive for mini-core myopathy |
| 28 | 1 | 47 | 0 | open | mild variation | + | 0 | 0 | 0 | 0 | T1 | n.a. | n.a. | n.a. | n.a. | T1, 60% | 0 | n.a. | 0 | 0 | small zone with Z-band alterations | non | non | Fibertype disproportion and small myopathic changes. |
| 29 | 1 | 27 | 0 | open | normal | + | 0 | 0 | 0 | 0 | T2 | n.a. | n.a. | n.a. | n.a. | T2 normal | 0 | 0 | 0 | 0 | cores | rods + | no | Core rod myopathy |
| 30 | 1 | 39 | 0 | open | minimal variation and increase of internal nuclei | + | + | i and c | 0 | 0 | T2 | n.a. | n.a. | n.a. | n.a. | T2 normal | 0 | n.a. | 0 | 0 | minimal myofibrilic changes, no cores | non | non | Minimal myofibrial changes |
| 31 | 1 | 41 | 0 | open | necrotic fiber, variation, increase of internal nuclei | ++ | ++ | mostly c | ++ | 1 necrotic fiber | T2 | n.a. | n.a. | n.a. | n.a. | T2 (clumping of T1) | unOS | n.a. | 0 | 0 | minicores | n.a. | n.a. | Small changes and minicores (in EM) |
| *MH nmean age* |  | 37 | 8 |  |  |  |  |  |  |  |  |  |  |  |  |  |  |  |  |  |  |  |  |  |
| ***RHABDOMYOLYSIS*** | | | | | | | | | | | | | | | | | | | | | | | | |
| 32 (M of 33) | 1 | 45 | 0 | needle | moderate variation and increase of internal nuclei | ++ | + | i | + | 0 | T2 | 29 | 108 | 10 | 88 | 24 | unOS | 0 | + | 0 | small zone with Z-band alterations | n.a. | n.a. | Aspecific myopathic changes |
| 33 | 1 | 15 | 0 | needle | mild variation | + | 0 | 0 | 0 | 0 | T2 | 30 | 70 | 50 | 80 | 45 | 0 | 0 | 0 | droplets | some fibers with alterations, not specific. | non | non | Small aspecific changes |
| 34 | 0 | 27 | 0 | needle | moderate variation and increase of internal nuclei | + | + | i | 0 | 0 | T2 | 30 | 105 | 25 | 90 | 49 | 0 | 0 | 0 | prominent droplets | fat-droplets | non | non | Myopathy with prominent lipid droplets |
| 35 | 0 | 61 | 1 | needle | variation and necrotizing fibers | + | + | i | 0 | + | T2 | 22,5 | 102 | 23 | 89 | 40 | cores | 2 | 0 | 0 | 4 x central cores, 1 x core | non | T1 and T2 | Core myopathy (not classic) |
| 36 | 1 | 16 | 0 | needle | moderate variation and many central internal nuclei | ++ | ++ | central | +++ | + | T1 | 55 | 120 | 55 | 125 | 55 | 0 | 0 | 0 | prominent droplets | larger mitochondria, some myelinfigures , degenerated fibers | non | non | Myopathic changes moderate fibervariation and internal nuclei |
| 37 | 1 | 24 | 0 | needle | mild variation and increase of internal nuclei | + | + | i and C | + | 0 | T2 | 30 | 115 | 35 | 105 | 45 | 0 | 0 | 0 | 0 | larger mitochondria, No cores | non | non | Small aspecific changes |
| 38 | 1 | 41 | 0 | open | mild to moderate variation and many internal nuclei | + | ++ | i | ++ | 0 | T2 | 65 | 111 | 57 | 119 | 30 | 0 | 0 | 0 | 0 | n.a. | n.a. | n.a. | Aspecific myopathic changes |
| *2nd biopsy* | *1* | *56* | *0* | *open* | *mild to moderate variation and many internal nuclei* | *+* | *++* | *i* | *+++* | *splitted fiber* | *T2* | *50* | *108* | *54* | *108* | *31* | *ringfiber* | *0* | *0* | *0* | *n.a.* | *n.a.* | *n.a.* | *Aspecific myopathic changes* |
| 39 | 0 | 39 | 0 | needle | moderate variation, mild increase of intenal nuclei | ++ | + | i and C | focal + | + | T1 | 30 | 64 | 48 | 76 | 55 | 0 | 2 | 0 | droplets | some ringmitochondria and crystal-like inclusions | non | non | Aspecific myopathic changes |
| 40 | 0 | 43 | 0 | needle | small fibers, increase of internal nuclei | + | 0 | 0 | - | + | T1 | 50,4 | 108,4 | 35 | 100 | 55 | 0 | 2 | 0 | droplets | normal | non | non | Small aspecific changes |
| 41 | 0 | 33 | 0 | needle | mild variation and small quantity of internal nuclei | + | + | i and C | (in some fiber) + | 0 | T2 | 16 | 69 | 17 | 66 | 45 | 0 | 0 | 0 | droplets | 2 times a small structural alteration of myofibrils, 1 degenerated cell | 2 x small structural myofibrillic changes | non | Small aspecific changes |
| 42 | 0 | 20 | 0 | open | normal | 0 | 0 | 0 | 0 | 0 | T2 | n.a. | n.a. | n.a. | n.a. | n.a. | 0 | 0 | 0 | 0 | n.a. | n.a. | n.a. | Normal |
| 43 | 0 | 5 | 0 | open | Very mild variation. | + | 0 | 0 | 0 | 0 | T1 | n.a. | n.a. | n.a. | n.a. | 75 | 0 | 0 | 0 | 0 | n.a. | n.a. | n.a. | Fibertype disproportion |
| 44 | 0 | 7 | 0 | open | Very mild variation. Single lymphocytic inflammatory focus/necrotic fiber | + | 0 | 0 | 0 | 1 necrotic fiber | T1 | n.a. | n.a. | n.a. | n.a. | 60 | unevenness of staining | 0 | 0 | 0 | possible minicore, Z-line streaming | non | non | Fibertype disproportion and a necrotic fiber |
| 45 | 0 | 44 | 0 | open | very prominent staining of peripheral mitochondria in most fibres, 4 ring fibres were noted, mild increase in sarcolemmal labelling of utrophin, Acid phosphatase: there is some increase in staining at the periphery of fibres with a pattern suggesting lipofuscin deposition. ? Target fibres | ++ | 0 | 0 | 0 | 0 | T1, no type IIb fibers | 50 | 90 | 50 | 90 | T1, no type IIb fibers | multiple ringfibers | 0 | 0 | 0 | n.a. | n.a. | n.a. | Ring fibres, increased mito in many fibres, type 1 fibre predominance |
| 46 | 1 | 26 | 0 | open | Acute widespread fibre necrosis with little evidence of fibre regeneration consistent with acute rhabdomyolysis | ++ | 0 | 0 | 0 | 0 | T2 | 60 | 100 | 60 | 100 | T2 normal | marked disruption of the architecture of necrotic fibres; viable fibres appeared normal | 0 | 0 | prominent droplets | n.a. | n.a. | n.a. | Acute fibre necrosis consistent with rhabdomyolysis |
| 47 | 0 | 13 | 0 | open (hamstrings) | Rare atrophic fibres including very small pin-prick type fibres and rare nuclear bag fibres are found | + | 0 | 0 | 0 | 0 | T2 | 40 | 90 | 40 | 90 | T2 normal | unevenness of oxidative staining | 0 | 0 | 0 | n.a. | n.a. | n.a. | Small aspecific changes, occasional small neonatal myosin positive pin-prick type fibres) |
| *2nd biopsy* | *0* | *18* | *1* | *open* | *Only report available for review* |  |  | *0* |  |  |  |  |  |  |  |  | *minicores were reported by pathologist in referring centre* |  |  |  | *n.a.* |  |  | *Mini cores* |
| 48 | 0 | 34 | 0 | open | occasional subsarcoleman droplets (? Artefacts), many fibres demonstrate a prominent mitochondrial punctate pattern | ++ | 0 | 0 | 0 | 0 | T2 | 50 | 100 | 50 | 100 | T2 normal | 0 | 0 | 0 | 0 | n.a. | n.a. | n.a. | Occasional scattered very small pin-prick fibres. Some droplets stained for PAS (artefacts?) |
| 49 | 1 |  | 0 | open | occasional atrophic fibres and several large hypertrophic fibres. There are rare nuclear bag fibres, but atrophic angulated fibres are not a feature. There is minimal patchy increase in connective tissue within endomysium. There are occasional split fibres. MIld increase in connective tissue is confirmed in gomori staining. | ++ | + | central |  | splitted fibers | T2 |  |  |  |  | T2 normal | unOS | 0 | 0 | droplets | n.a. | n.a. | n.a. | Mild aspecific changes, increased endomysial connective tissue, mild excess of lipid droplets |
| 50 | 0 | 28 | 0 | open | normal/very mild variation | 0 | 0 | 0 | 0 | 0 | T2 | 44 | 87 | 40 | 93 | <50 | 0 |  | 0 | 0 | n.a. | n.a. | n.a. | essentially normal |
| *RM mean age* |  | 30 |  |  |  |  |  |  |  |  |  |  |  |  |  |  |  |  |  |  |  |  |  |  |
| *Mean age* |  | *34* |  |  |  |  |  |  |  |  |  |  |  |  |  |  |  |  |  |  |  |  |  |  |
